# Extracellular Matrix in Heart Disease: Focus on Circulating Collagen Type I and III Derived Peptides as Biomarkers of Myocardial Fibrosis and Their Potential in the Prognosis of Heart Failure: A Concise Review

**DOI:** 10.3390/metabo12040297

**Published:** 2022-03-28

**Authors:** Asparuh Nikolov, Nikola Popovski

**Affiliations:** 1 Cardiovascular Research Working Group, Division of Medicine, Institute for Scientific Research, Medical University-Pleven, 5800 Pleven, Bulgaria; 2 Clinic of Obstetrics and Gynaecology, Department of Obstetrics and Gynaecology, University Hospital Pleven, Medical University-Pleven, 5800 Pleven, Bulgaria; n_popovsky@abv.bg

**Keywords:** extracellular matrix, collagen type I and III derived peptides, biomarkers, heart failure, myocardial fibrosis

## Abstract

Accumulating evidence indicates that two major proteins are responsible for the structural coherence of bounding cardiomyocytes. These biomolecules are known as myocardial fibrillar collagen type I (COL1) and type III (COL3). In addition, fibronectin, laminin, fibrillin, elastin, glycoproteins, and proteoglycans take part in the formation of cardiac extracellular matrix (ECM). In physiological conditions, collagen synthesis and degradation in human cardiac ECM are well-regulated processes, but they can be impaired in certain cardiovascular diseases, such as heart failure (HF). Myocardial remodeling is part of the central mechanism of HF and involves cardiomyocyte injury and cardiac fibrosis due to increased fibrillar collagen accumulation. COL1 and COL3 are predominantly involved in this process. Specific products identified as collagen-derived peptides are released in the circulation as a result of abnormal COL1 and COL3 turnover and myocardial remodeling in HF and can be detected in patients’ sera. The role of these products in the pathogenesis of cardiac fibrosis and the possible clinical implications are the focus of numerous investigations. This paper reviews recent studies on COL1- and COL3-derived peptides in patients with HF. Their potential application as indicators of myocardial fibrosis and prognostic markers of HF is also highlighted.

## 1. Introduction

Heart failure (HF) has been recognized as a worldwide health burden that affects approximately 40 million people globally [1]. It has been estimated that the incidence of HF in adults is about 2%, and the rate rises to 6–10% over the age of 65 [2]. For those older than 75 years, the rate is more than 10% [3]. In addition, because of the increased life expectancy and risk factors such as hypertension, diabetes, dyslipidemia, and obesity, the morbidity rate is also expected to rise [4]. It has been reported that in people over the age of 65, heart failure is the leading cause of hospitalization.

Based on left ventricular ejection fraction (LVEF) values, the European Society of Cardiology (ESC) divides HF into three types: with preserved ejection fraction (HFpEF), characterized by LVEF ≥ 50%; mid-range (HFmrEF), with LVEF of 40–49%; and with reduced ejection fraction (HFrEF), with LVEF < 40% [5]. Considering the underlying etiologies, demographics, comorbidities, and responses to therapy, differentiation of HF according to LVEF has significant practical value.

## 2. Type I and Type III Collagen Characteristics

Collagen (COL) is the main fibrous protein in human ECM, accounting for more than one-third of total protein content in the organism [6]. Practically, it is present in all body systems containing connective tissue. Collagen is responsible for the strength and stability of the cytoskeleton and regulates normal cell and tissue development [7]. Different COL types form collagen fibers, so they represent a heterogeneous mix. However, in any given tissue, a certain type of collagen usually prevails [8].

Collagen type I is a fibrillar protein that makes up a large part of the structure of the interstitial membrane. It is known as the most common type of collagen, and is an important structural component of many tissues. COL1 can be found in almost all connective tissue structures. It is a structural protein found in bones; skin, tendons; ligaments; sclera; corneas; and blood vessels, as well as other tissues. It is a component aligned in fibers, thus forming a structural-mechanical scaffold (matrix) for bones; skin; tendons; corneas; blood vessel walls; and other connective tissues. The dominant isoform of COL1 is heterotrimers with two α1 (I) and one α2 (I) chain. In fetal tissues and some fibrous lesions, homotrimers with three α1 (I) chains have been discovered [9]. The homotrimeric isoform is known to be less susceptible to cleavage by collagenases, which may clarify its accumulation and functional role in tumors and fibrotic lesions [10].

Collagen type III has a unique molecular structure. A long protein chain is responsible for its tensile stiffness and the biomechanical characteristics of tissues. This contributes to the specific ECM properties when this type of collagen predominates. It is an important component of reticular fibers in the interstitial tissue of the lungs, liver, heart, and vessels [11].

Collagen type III is made up of only one collagen α chain. COL3 is a homotrimer made up of three α1 (III) chains overlapped in a right triple helix. It is produced by fibroblasts and other mesenchymal cells and plays an important role in inflammatory conditions such as lung damage, liver diseases, and renal and vascular fibrosis. Consequently, COL3 and COL1 are both important components of the myocardial ECM [12]. Today, immunological markers based on collagen type I and III turnover have been extensively investigated for detection of cardiac fibrosis.

## 3. Cardiac Extracellular Matrix: Structure and Function

The extracellular matrix (ECM) is made up of a fibrillar network along with a basement membrane, proteoglycans, and fibrous proteins such as fibronectins, collagens, elastins, fibrillins, and laminins [13]. Together they maintain the structural coherence of bounding cells, ensuring stability. The ECM has also been associated with the transmission of important biochemical signals, which are crucial for normal tissue development. ECM is present in all tissues, but each organ has a unique distribution of matrix components [14]. For example, cardiac ECM is primarily composed of collagen type I (85%) and III (11%). The traditional concept of myocardial ECM was that it was an inert mechanical scaffold providing structural cardiac integrity. Nowadays, it is considered as a dynamic network with important metabolic activity and many complex functions, such as regulation of molecular signaling; cell proliferation; differentiation, migration; adhesion; and protein interactions. Moreover, it also regulates myocardial remodeling in normal and pathological conditions. Therefore, cardiac ECM plays a fundamental role in maintaining cardiovascular homeostasis [15]. With the accumulation of additional knowledge about the structure and function of heart ECM, cardiac fibroblasts have been described as the primary source of myocardial COL1 and COL3 peptides. It can be concluded that they are the main cells producing collagen in the heart [14,15].

**Figure 1:**
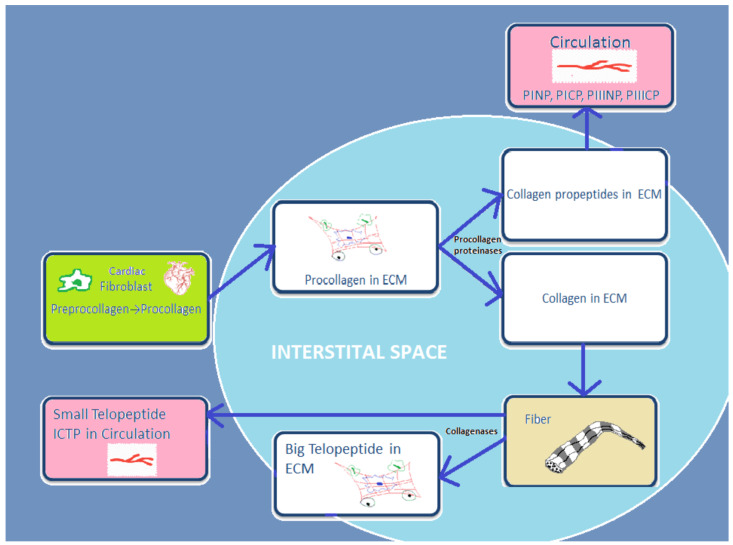
Schematic presentation of basic stages from process of synthesis and degradation of collagen I and III.

Cardiac fibroblasts are the major heart cells producing COL1 and COL3. Fibrillar collagen is initially synthetized as a procollagen, which is then split by specific proteinases into carboxy (C)- and amino (N)-terminal propeptides: N-terminal propeptides of COL1 and COL3 (PINP and PIIINP) and C-terminal propeptides (PICP and PIIICP). Thereafter, they are secreted in the circulation. Since propeptides are split, the triple helix chain “will form big collagen fibers with other collagen chains” [16]. Collagenases MMP-1, -8, and -13 degrade these collagen fibers, and telopeptides are formed during this process. Then the small telopeptides of collagen type I (ICTP, 12 kDa) are released into the circulation [17]. The big telopeptides go through spontaneous denaturation in nonhelical derivatives [18]. Subsequently, gelatinases MMP-2 and -9 completely degrade them into inactive fragments (Figure 1).

## 4. General Concepts of Abnormal Cardiac Extracellular Matrix Changes in Heart Failure

ECM is a dynamic structure that plays a crucial role in the development and progression of many cardiovascular diseases. Accumulating data indicate that fibrosis is observed in different cardiovascular diseases (CVDs). HF is an example of abnormal collagen accumulation, which pathologically increases myocardial stiffness and impairs heart contractile properties. Several CVDs, including hypertension; coronary artery disease; valvular disease; and arrhythmias, are considered to be leading causes of HF. A link has been found between cardiac remodeling and the development of HF [19]. Cardiac remodeling is defined as “a group of molecular, cellular and interstitial changes that manifest clinically as alterations in the size, mass, geometry and function of the heart after a stressful stimulus.” [20,21]. This process can be triggered by “ischemia (myocardial infarction), inflammation (myocarditis), hemodynamic overload (workload by volume or pressure) and neurohormonal activation” [22,23,24].

Paradoxically, cardiac remodeling is thought to be both an adaptive and a maladaptive process. Initially, cellular changes in the heart structure, such as myocyte hypertrophy, necrosis, and apoptosis, occur and then extracellular matrix deposition of fibrillar collagen increases (a process often defined as “myocardial fibrosis”) [25,26,27]. This has been related to impaired collagen metabolism, manifesting as accelerated synthesis and accumulation of COL1 and COL3 in the myocardium [28,29]. Accordingly, collagen degradation is slowed down and heart function is inevitably altered in the later stages of cardiac remodeling [30].

## 5. Basic Underlying Mechanisms of Myocardial Fibrosis in Heart Failure: Role of Impaired Type I and III Collagen Turnover

There is a large body of evidence regarding the accelerated myocardial accumulation of fibrillar collagen in heart failure. Early research on HF showed that various MMPs are present in the myocardium of patients with chronic heart failure [31]; since then, there has been ongoing enthusiasm for the routine application of COL1 and COL3 as biological markers for assessing cardiac tissue remodeling and myocardial fibrosis. This is true for both laboratory models and clinical studies [32]. In important experiments, Douglas et al.’s [31] and Alla’s [32] findings suggested delayed degradation of collagen in patients with chronic HF, thereby contributing to the mechanism of myocardial fibrosis development.

Increased serum levels of COL1 and COL3 synthesis biomarkers (PICP, PINP, PIIINCP, PIIINP) and decreased serum levels of the COL1 degradation biomarker (ICTP) have been linked to myocardial collagen deposition and fibrosis [33,34,35]. According to these findings, the equilibrium between synthesis and degradation of cardiac collagen is disrupted in heart disease [31,32]. Importantly, heart failure is an example of CVD disease that presents with altered collagen turnover [36,37].

**Figure 2:**
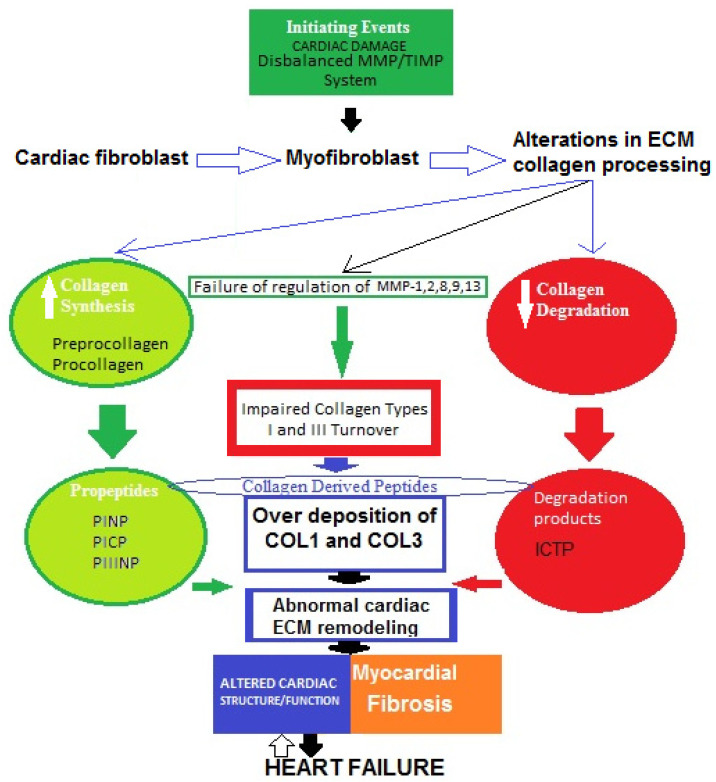
Possible schematic pattern illustrating eventual mechanisms of impaired collagen I and III turnover leading to myocardial fibrosis in heart failure.

A widely held concept regarding the pathogenesis of myocardial fibrosis is that cardiac lesion is considered to be an initiating event [38]. Thus, the MMP/TIMP system fails and the degradation activity of MMP-1, -2, -8, -9, and -13 is disturbed. As a result, fibroblasts in the heart are hyperactivated and transdifferentiated into myofibroblasts, which increase the production of collagen types I and III, then degradation processes decrease and abnormal collagen deposition in myocardium occurs [39,40]. Therefore, impaired collagen turnover abnormally affects the remodeling of cardiac ECM, and COL1-/COL3-derived peptides are released into the circulation. This can trigger an incessant vicious cycle of COL1/COL3 over-deposition and consequent suppressed degradation.All of these processes might contribute tothe development of cardiac fibrosis in heart failure. However, the molecular mechanisms of the genesis and progression of myocardial fibrosis are not yet fully clear (Figure 2) [39,40,41,42,43,44,45].

The four major collagen-derived peptides of research interest nowadays are the N-terminal propeptide of collagen type I (PINP), the N-terminal propeptide of collagen type III (PIIINP), the C-terminal propeptide of collagen type I (PICP), and the C-terminal telopeptide of collagen type I (ICTP). PINP and PICP indicate collagen type I synthesis, PIIINP reflects collagen type III synthesis, and ICTP represents collagen type I degradation. Matrix metalloproteinases (MMPs) and tissue inhibitors of MMPs (TIMPs) are other molecules produced by cardiac fibroblasts. MMPs are proteolytic enzymes that degrade ECM proteins and TIMPs are MMP inhibitors, maintaining a fine balance between synthesis and degradation processes. Thus, both MMPs and TIMPs are regulatory proteins essential for ECM homeostasis.

## 6. Collagen Type I and III Derived Peptides as Biomarkers in Heart Failure

### 6.1. Detection of Circulating PIIINP

PIIINP has been described as a parameter of COL3 synthesis. A large part of serum PIIINP is produced during the extracellular conversion of pro-COL3 to COL3 by procollagen amino-terminal proteinase. The concentration of PIIINP in serum has been associated with the myocardial area fractions of their tissue analogues [46]. In an important investigation, Klappacher et al. [47] examined the sera of patients with dilated cardiomyopathy (DCM) and found accelerated myocardial extracellular matrix turnover. Of note, PIIINP also has an effect on the determination of risk and prognosis. Additionally, it was found that, in HF patients treated with spironolactone, a decrease in collagen volume fraction was found to occur with a decrease in serum PIIINP levels [48]. Most recently, in a study involving patients with acute heart failure (AHF), Nagao et al. investigated the time-dependent prognostic utility of PIIINP and type IV collagen 7S (P4NP 7S)). P4NP 7S significantly decreased during hospitalization, whereas PIIINP did not. The authors concluded that there was no correlation between increased PIIINP levels and significant excess risk for both 90-day and 365-day outcomes [49]. 

Several authors have described that serum PIIINP is associated with outcomes in HF of different causes, regardless of EF. For example, Zannad et al. [50] reported decreased survival rates in patients with HFrEF with a PIIINP cut-off point of >3.85 μg/L, whereas the data of Klappacher et al. showed a PIIINP cut-off point of >7 μg/L, with the clarification that the latter’s patients all had DCM (33 idiopathic and 8 ischemic cases). It appears that patients with HF and dilated hypertrophic cardiomyopathy (HCM) have significantly higher serum PIIINP levels than healthy controls. Hypertensive patients with HFpEF also showed significantly higher serum PIIINP concentrations than both hypertensive HFrEF and HFmrEF patients [51]. With regard to HFmrEF, there is only one study with such patients, which shows decreased survival with PIIINP > 4.7 μg/L [52]. Elevated PIIINP levels are related to decreased survival rates in both HF and DCM [52,53]. Of note, serum PIIINP levels are also significantly higher in patients with acute myocardial infarction, and a PIIINP cut-off point of >5 μg/L was determined as an independent predictor of cardiac death and in-hospital development of congestive HF [54].

Recently, Nikolov et al. found significantly higher circulating levels of serum PIIINP in patients with HFmrEF and coronary artery disease (CAD) with left ventricular hypertrophy (LVH) than in patients with HFmrEF and CAD without LVH [55]. Another study, by Lee et al. investigated the link between PIIINP, left ventricular end-diastolic pressure (LVEDP), and cardiovascular events in patients with acute coronary syndrome (ACS). The authors reported that PIIINP is very useful in evaluating left ventricular end-diastolic pressure. Moreover, they noted that PIIINP is related to cardiac mortality and revascularization. Hence, they provided not only an additional means of evaluating patients with ACS, but also important treatment directions [56]. Eastell et al. have described that serum PIIINP is associated with severity of HF of different causes, regardless of EF [57].

Barasch et al. found that both HFrEF and HFpEF were associated with significantly elevated amino-terminal peptide of pro-COL3 [58].

Zile et al. reported increased PIIINP levels in HF patients [59]. Previously, Schwartzkopff et al. described PIIINP as an independent predictor of HF mortality [60]. In their study, Michalski et al. examined HFrEF vs. HFpEF patients and reported that PIIINP showed a strong negative correlation with left ventricular strain [61]. Zile’s results were further confirmed by the large Multi-Ethnic Study of Atherosclerosis (MESA) in 2018. MESA evaluated the predictive value of serum PIIINP and ICTP in 3187 patients with HF who were divided into two subgroups, with and without preserved ejection fraction [62]. MESA concluded that elevated levels of circulating ICTP and PIIINP were related to the incidence of HFpEF, but not HFrEF. Table 1 presents the findings from studies determining PIIINP levels in patients with heart failure.

### 6.2. Detection of Circulating PINP

Procollagen type I propeptides are products derived from the COL1 molecule. This precursor is composed of a brief signal sequence and two peptides, amino-terminal propeptide (PINP) and carboxy-terminal propeptide (PICP). Propeptide extensions are later removed by specific proteinases and can be found in the circulation. Their serum levels indicate the COL1 synthesis rate [65]. PINP was cited as an indicator of COL1 synthesis. Interestingly, PINP concentration is not significantly different in patients with HCM [66]. According to Martos et al. this is also valid for hypertensive patients with or without diastolic HF, compared with healthy controls.

In order to estimate the predictive value of PINP, PIIINP, and ICTP in HF, Dupuy et al. examined these parameters together with two mediators of cardiac fibrosis, galectin-3 and soluble suppression of tumorigenicity-2 protein (sST2). The authors concluded that a multi-marker strategy demonstrated the greatest prognostic improvement when “attained with the combined addition of ICTP/PIIINP ratio and sST2 highlighting the potential role of fibrosis pathways in risk stratification” [67].

### 6.3. Detection of Circulating PICP

PICP acts as an indicator of COL1 synthesis. Serum C-terminal propeptide of pro-COL1 is produced during the extracellular conversion of pro-COL1 into COL1. This reaction is catalyzed by the enzyme procollagen C-terminal proteinase [46]. Querejeta et al. provided evidence that a net release from the heart into the circulation occurs in HF [68]. In 2021, He et al. studied subjects with suspected HF from the HOMAGE-HULL sub-cohort. They used ELISA to quantify the collagen synthesis biomarker PICP and collagen degradation biomarkers ICTP and matrix metalloproteinase (MMP-1). They detected a significantly positive association between heart failure death and ICTP and MMP-1, but not PICP [69].

This supports the suggestion that systemic PICP has a cardiac origin. Additionally, serum PICP levels were related to collagen volume fraction [70,71,72].

PICP circulation has been the subject of many studies. For example, Lopez et al. [73] concluded that PICP levels decreased in patients with hypertensive heart failure who receivedtorasemide treatment. Löfsjögård et al. reported that serum PICP is associated with HFrEF severity [74], whereas Krum et al. [75] noted that PICP is related to mortality in HFpEF. In 2017, Löfsjögård et al. [76] reported that PICP is linked with mortality in HFrEF. Of interest, Flevari et al. found that the serum PICP-to-PIIINP ratio is related to malignant ventricular arrhythmogenesis in HF [77]. When analyzing these data, it can be observed that PICP concentrations are significantly increased in more studies of patients with HFpEF than HFrEF. Except in the studies of Alla et al. [32] and Plaksej et al. [63], patients with HF have been shown to have higher serum PICP levels than controls. Both serum and coronary PICP are positively correlated with the myocardial collagen content [78]. The investigation by Quaretta et al. is the only research involving subjects with HFmrEF, and it reports that the abnormal increase in cardiac COL1 synthesis and deposition may play an important role in the augmentation of myocardial fibrosis in HF from hypertensive origin [79]. Similarly, analysis of PICP in patients with HCM and in patients with cardiomyopathies shows elevated serum PICP in patients with mild to moderate dilated cardiomyopathy [64,80,81].

Ruiz-Ruiz et al. examined the relationship between serum levels of propeptide of procollagen type I and outcomes in 111 patients with decompensated heart failure, considering death from any cause or due to heart failure and readmission as primary endpoints. Serum levels of propeptide of procollagen type I were significantly higher among patients who experienced either endpoint during follow-up. The investigators suggested that “a single serum measurement of propeptide of procollagen type I may possibly have prognostic value in patients with decompensated heart failure”. Accordingly, patients with higher levels of propeptide of procollagen type I at decompensation are at a higher risk of death or readmission during follow-up [82].

In another study, Raafs et al. tested whether the combination of blood PICP levels and late gadolinium enhancement (LGE) in cardiac magnetic resonance (CMR) could provide additional prognostic information in idiopathic dilated cardiomyopathy. Using those methods plus invasive endomyocardial biopsy (EMB) and collagen volume fraction, (CVF), the authors quantified fibrosis in 209 DCM patients. The major conclusion was that a “combination of myocardial fibrosis at CMR and circulating PICP levels provides additive prognostic value accompanied by a pro-fibrotic and pro-inflammatory transcriptomic profile in DCM patients with LGE and elevated PICP” [83]. Table 2 represents the findings from the studies determining PICP levels in patients with heart failure.

### 6.4. Detection of Circulating ICTP

ICTP is an indicator of COL1 degradation. Studies have shown that ICTP concentrations are increased in both HFrEF and HFpEF patient groups. Patients with DCM and HCM also express elevated serum ICTP levels. Previously, Klappacher et al. indicated that serum ICTP is positively correlated with myocardial collagen content. Later, Kitahara et al. concluded that ICTP is a predictor of mortality at levels > 7.6 μg/L [84].

Plaksej et al. evaluated serum ICTP levels in hypertensive patients with HF and reported increased concentrations in those with New York Heart Association (NYHA) class IV HF [63]. On the other hand, Barasch et al. did not find an association between ICTP and HFrEF or HFpEF. Manhenke et al. [85] demonstrated that ICTP is an independent predictor of total and cardiovascular mortality in patients with an acute myocardial infarction.

Another intriguing approach in the search for HF indicators is to assess the ratio of ECM components. For example, the ICTP-to-MMP-1 ratio was recently studied by López et al. for evaluation as a novel biomarker. It has been theorized that, since collagen cross-linking modifies the resistance of collagen fiber to MMP degradation, the higher the cross-linking of COL1 fibers, the lower the cleavage of ICTP by MMP-1. Consequently, the serum ICTP-to-MMP-1 ratio is inversely correlated with myocardial collagen cross-linking [86]. López et al. also found that the ICTP-to-MMP-1 ratio is independently associated with the risk of HF hospitalization. Furthermore, Ravassa et al. provided evidence for an integrative strategy combining the ICTP-to-MMP-1 ratio with PICP measurement. With this test, a decreased ICTP-to-MMP-1 ratio and elevated PICP concentrations indicated HF patients with the highest risk [87].

Similarly, Zile et al. detected increased ICTP levels, but in HFpEF patients. Schwartzkopff et al. found increased ICTP levels in HFmrEF patients. Ristelli et al. and Cornelissen et al. also analyzed connective tissue metabolites in human serum [88,89]. Batlle et al. reported elevated ICTP concentrations and increased risk of a clinical event [90]. In 2018, MESA demonstrated high levels of circulating ICTP. Table 3 presents findings from the studies determining ICTP levels in patients with heart failure.

Curiously, in recent years, only two studies have assessed myocardial fibrosis in patients with severe aortic stenosis and HF symptoms. First, Echegaray et al. analyzed the myocardial collagen volume fraction from left ventricular free wall transmural biopsies and suggested that in symptomatic HFpEF patients with severe aortic stenosis, diastolic dysfunction is related to intensified nonmysial deposition of COL1. This leads to increased ECM stiffness [91]. Later, Park et al. reported that extracellular volume is more closely related to predictions of clinical outcomes in severe aortic stenosis than native T1 mapping sequences generated from cardiac magnetic resonance or global longitudinal strain measured by speckle-tracking echocardiography, although the result was not statistically significant [92]. As for atrial fibrillation (AF), Ravassa et al. examined circulating COL1 biomarkers in samples from both HF patients and patients referred for AF ablation. The researchers found that serum indicators of intensified myocardial COL1 cross-linking and deposition are associated with higher AF prevalence, incidence, and recurrence after ablation [93].

Beyond the diagnosis of myocardial fibrosis, Ravassa et al., Hinderer et al., Bing et al., and Lopez et al. [39,40,41,42] highlighted the importance of circulating biomarkers of fibrillar COL1- and COL3-derived peptides as valuable non-invasive prognostic tools for determining clinical outcomes of patients with HF.

## 7. Limitations of and Future Prospects for the Application of COL1 and COL3 Derived Peptides as Biomarkers of Myocardial Fibrosis and Prognostic Indicators of Heart Failure

In light of the above-mentioned reports, a question of great interest arises: is it possible that circulating COL1 and COL3 peptides mark the development of myocardial fibrosis and even predict a prognosis of heart failure? Our current approach to collagen-derived peptides as parameters of COL1 and COL3 metabolism assumes that they have the potential to be used in routine clinical settings in the near future. Despite this argument, the studies discussed here contain some controversial findings and limitations. Several remarks uncover gaps in the possible application of PINP, PICP, PIIINP, and ICTP as biomarkers of myocardial fibrosis and prognostic indicators of heart failure.

Alterations in COL1 and COL3 metabolism are likely to occur early in the course of heart failure, even before clinical manifestation of HF. A large proportion of the cited research involved patients with established HF; therefore, it cannot be determined whether the change in collagen-derived peptide levels developed before or after the onset of HF. To better understand the link between these markers and concurrent HF, it would be appropriate to closely monitor a group of subjects with clinically unmanifested HF and test them for abnormal changes in COL1 and COL3 turnover biomarkers. Moreover, different studies involved diverse patient groups, such as patients with HFpEF [51,63,66,68,78,85,87]; HFmrEF [52,55,62,86]; or HFrEF [32,50,67,69,78,87]; or did not specify any subgroups [54,61,71,73,78,80,81,85,88,89]. Some studies investigated patients with DCM [47,53,77,86] or HCM [74,75]. The diversity of cohorts makes it very difficult to perform a head-to-head comparison of the results obtained from these experiments.

Whether COL1 and COL3 turnover indicators alone might provide enough prognostic information, or a combination model with other known cardiovascular markers added such as natriuretic peptides and galectins would be useful, is another exciting question. Furthermore, image tests such as contrast CMRs are also used for the detection of myocardial fibrosis. In this regard, it may be more reasonable to use an integrated model that combines blood markers and image tests [77], rather than just one of them, for better prognosis of heart failure. This theory requires additional investigation.

Considering the studies covered in this review, some major differences can be observed between the methods applied for detection of serum markers of COL1 and COL3 metabolism. For non-invasive assessment and measurement of concentrations, researchers used radioimmunoassay [32,34,47,53,62,63,68,71,72,77,78], ELISA [49,52,55,59,60,61,65,66,67,69,75], and both of these [51]. Therefore, some differences can be observed between the cut-off points for detection of serum levels of COL1 and COL3 peptides. This suggests that not only the absolute parameter levels, but also their ratios [60,63,69,70,71,72,73,74,75,76,77,78,79,80,81], may be relevant in the assessment of myocardial remodeling and the risk of HF hospitalization.

Another important feature that should also be pointed out is the etiology of heart failure, because it can affect collagen turnover in a variety of ways. Hypertension, for instance, is associated with cardiac myocyte hypertrophy and increased stiffness of the ventricular wall, implying an increase in collagen fibers. As a result, patients with underlying hypertension express higher levels of COL1 and COL3 synthesis markers such as PIIINP and PICP. The levels of serum collagen-derived peptides can also be affected by different patient characteristics, such as the type of HF treatment, comorbidities, age, and BMI. In addition, the heart failure functional class should also be taken into account. It is important to distinguish whether collagen peptides are measured in patients with NYHA class I-II or III-IV. Likewise, it is important to highlight whether studies include subjects with acute or chronic heart failure, because these conditions can affect the concentrations of circulating collagen peptides.

It is well known that cardiac remodeling is a continual process. Unfortunately, most studiesused a cross-sectional design and indicated COL1 and COL3 peptide concentrations at only a certain moment. Instead, these biomarkers should be determined in serial measurements at various time points. There is a need for larger, longitudinal investigations.

COL1- and COL3-derived peptides are not entirely cardiac-specific, and changes in their levels may represent a wide range of cardiovascular pathologies, including comorbidities. Fibrosis can affect many organs, and it is possible that impaired collagen type I and III turnover (manifested by elevated serum concentrations of these markers) may have either cardiac or extracardiac origin. Notably, collagen-derived peptides can also be produced by other organs, such as bone, liver, kidney, lung, etc. [83]. Currently, EMB with evaluation of collagen volume fraction represents the most reliable test for the diagnosis of myocardial fibrosis. However, EMB is an invasive method based on histological analysis, and non-invasive tools would be more appropriate for routine practice. CMR imaging is precise, but has a high price and requires trained personnel. That is why research is aimed at investigating new blood markers such as COL1- and COL3-derived peptides. Their serum levels have been reported to correlate with the collagen volume fraction. Although these peptides are not entirely cardiac typical, important relationships have been found between their blood concentration and histologically assessed myocardial collagen deposition, HF severity, and prognosis. In order to more completely determine the differences in leakage from organs other than the heart, more studies exploring cardiac collagen type I and III turnover are needed. They could possibly detect novel molecules as entirely myocardial specific COL1 and COL3 precursors, enzymes, or degradation products. It also remains to be clarified whether medicament treatment could affect the elimination of collagen peptides by the liver and kidneys, as well as the kinetics and dynamics of these processes in the cardiac extracellular matrix.

## 8. Conclusions

Based on the current understanding, myocardial fibrosis in heart failure is associated with impaired collagen type I and III turnover, abnormal cardiac ECM remodeling, and the development of structural and functional heart changes. The modification of myocardial collagen content is a crucial factor in these pathological processes. As a result, COL1- and COL3-derived peptides are released into the circulation. These biomolecules have promising potential as markers of myocardial fibrosis and could be useful in the prognosis of heart failure.

## Figures and Tables

**Table 1:** Serum PIIINP concentrations in patients with heart failure.

Authors	Heart Failure Type	Main Findings
Alla et al. [32]	HF vs. HHD with T2DM vs. HC	PIIINP levels were higher in HF and HHD with T2DM than HC PIIINP levels were higher in HF than HHD with T2DM
Barasch et al. [58]	HFrEF vs. HFpEF	Associated with HFrEF and HFpEF
Cicoira et al. [52]	HFmrEF, single arm	Decreased survival with PIIINP > 4.7 μg/L
Martos et al. [51]	HFpEF, single arm	Increased PIIINP
Plaksej et al. [63]	HF vs. HC	Increased levels in NYHA class III and IV
Zannad et al. [50]	HFrEF, single arm	Decreased survival with PIIINP > 3.85 μg/L
Zile et al. [59]	HHD and HFrEF vs. HC	Elevated PIIINP
Klappacher et al. [47]	DCM vs. HC	Decreased survival with PIIINP > 7 μg/L
Fassbach et al. [64]	HCM vs. HC	Increased PICP in patients with HCM
Michalski et al. [61]	HFrEF vs. HFpEF	Strong negative correlation of PIIINP with LV strains
Multi-Ethnic Study of Atherosclerosis (MESA) [62]	HFrEF vs. HFpEF	Elevated PIIINP
Nagao et al. [49]	Acute HF, single arm	High PIIINP did not correlate with significant excess risk for outcome
Sato et al. [53]	DCM, single arm	Elevated PIIINP levels associated with decreased survival rate
Poulsen et al. [54]	HF vs. HC	Increased PIIINP level > 5 μg/L is an independent predictor of cardiac death and in-hospital development of HF
Nikolov et al. [55]	HFmrEF vs. HC	Increased PIIINP
Schwartzkopff et al. [60]	DCM vs. HC	Independent predictors of mortality

NYHA, New York Heart Association; DCM, dilated cardiomyopathy; HF, heart failure not specified by ejection fraction; HFrEF, heart failure with reduced ejection fraction; HFmrEF, heart failure with mid-range ejection fraction; HFpEF, heart failure with preserved ejection fraction; HC, healthy control; T2DM, type 2 diabetes mellitus.

**Table 2:** Serum PICP concentrations in subjects with heart failure.

Authors	Heart Failure Type	Main Findings
Lopez et al. [78]	Torasemide-treated vs. furosemide-treated HF	Collagen volume fraction correlated with PICP
Querejeta et al. [68]	HHD with vs. without HF	Elevated PICP
Plaksej et al. [63]	HF vs. HC	Non-significant difference
Flevari [77]	HF, single arm	Relation between number of tachyarrhythmic episodes and PICP/PIIINP and ejection fraction
Ruiz-Ruiz [82]	HF, single arm	Higher PICP levels at decompensation correlated with higher risk of death or readmission
Martos et al. [51]	HFpEF, single arm	Increased PICP levels
Barasch et al. [58]	HFrEF vs. HFpEF	Associated with HFpEF
Alla et al. [32]	HF vs. HHD with T2DM vs. HC	Lower PICP and PINP in HHD with T2DM than HC
Schartzkopff et al. [60]	DCM vs. HC	Elevated serum PICP in patients with mild to moderate DCM
He et al. [69]	HF, single arm	PICP not associated with heart failure death
Löfsjögård et al. [74,76]	HFrEF, single arm	PICP associated with severity and mortality
Krum et al. [75]	HFpEF, single arm	PICP associated with mortality in HFpEF
Fassbach et al. [64]	HCM vs. HC	Increased PICP
Raafs et al. [83]	DCM, single arm	Elevated PICP
Lombardi et al. [81]	HCM vs. HC	Increased PICP

NYHA, New York Heart Association; HCM, hypertrophic cardiomyopathy; HHD, hypertensive heart disease; HF, heart failure not specified by ejection fraction; HFrEF, heart failure with reduced ejection fraction; HFmrEF, heart failure with mid-range ejection fraction; HFpEF, heart failure with preserved ejection fraction; HC, healthy control; T2DM, type 2 diabetes mellitus.

**Table 3:** Serum levels of ICTP in patients with heart failure.

Authors	Heart Failure Type	Main Findings
Plaksej et al. [63]	HF vs. HC	Elevated ICTP
Kitahara et al. [84]	HFrEF vs. HFpEF	Event-free point decreases when ICTP > 7.3 ng/mL
Barasch et al. [58]	HFrEF vs. HFpEF	Not related to HFpEF or HFrEF
Zile et al. [59]	HHD and HFrEF vs. HC	Elevated ICTP
Klappacher et al. [47]	DCM vs. HC	Increased mortality when ICTP > 7.6 μg/L
Schartzkopff et al. [60]	HFmrEF vs. HC	Increased ICTP levels
Batlle et al. [90]	HFrEF-single arm	Elevated ICTP and higher risk of a clinical event
MESA (Multi-Ethnic Study of Atherosclerosis) [62]	HFrEF vs. HFpEF	High levels of circulating ICTP
Ravassa et al. [87]	HF, single arm	Combination of low ICTP-to-MMP-1 ratio and high PICP identifies HF patients at highest risk of a clinical event
Lopez et al. [86]	HF, single arm	ICTP-to-MMP-1 ratio independently associated with risk of HF hospitalization

NYHA, New York Heart Association; DCM, dilated cardiomyopathy; HF, heart failure not specified by ejection fraction; HFrEF, heart failure with reduced ejection fraction; HFmrEF, heart failure with mid-range ejection fraction; HFpEF, heart failure with preserved ejection fraction; HC, healthy control; MMP-1, matrix metalloproteinase-1.

## Data Availability

Not applicable.
